# Social convergence of gut microbiomes in vampire bats

**DOI:** 10.1098/rsbl.2021.0389

**Published:** 2021-11-03

**Authors:** Karthik Yarlagadda, Imran Razik, Ripan S. Malhi, Gerald G. Carter

**Affiliations:** ^1^ Department of Anthropology, University of Illinois at Urbana-Champaign, Urbana, IL, USA; ^2^ Program in Ecology, Evolution and Conservation Biology, University of Illinois at Urbana-Champaign, Urbana, IL, USA; ^3^ Carl R. Woese Institute for Genomic Biology, University of Illinois at Urbana-Champaign, Urbana, IL, USA; ^4^ Department of Evolution, Ecology and Organismal Biology, The Ohio State University, Columbus, OH, USA; ^5^ Smithsonian Tropical Research Institute, Balboa, Ancón, Panama

**Keywords:** vampire bat, microbiome, social network

## Abstract

The ‘social microbiome’ can fundamentally shape the costs and benefits of group-living, but understanding social transmission of microbes in free-living animals is challenging due to confounding effects of kinship and shared environments (e.g. highly associated individuals often share the same spaces, food and water). Here, we report evidence for convergence towards a social microbiome among introduced common vampire bats, *Desmodus rotundus*, a highly social species in which adults feed only on blood, and engage in both mouth-to-body allogrooming and mouth-to-mouth regurgitated food sharing. Shotgun sequencing of samples from six zoos in the USA, 15 wild-caught bats from a colony in Belize and 31 bats from three colonies in Panama showed that faecal microbiomes were more similar within colonies than between colonies. To assess microbial transmission, we created an experimentally merged group of the Panama bats from the three distant sites by housing these bats together for four months. In this merged colony, we found evidence that dyadic gut microbiome similarity increased with both clustering and oral contact, leading to microbiome convergence among introduced bats. Our findings demonstrate that social interactions shape microbiome similarity even when controlling for past social history, kinship, environment and diet.

## 1. Introduction

The ‘social microbiome’—defined as the collective microbial community of an animal social network—can fundamentally shape the costs and benefits of group living [1]. A social microbiome is measured by sampling microbes across the social network that provides a reservoir of both pathogens and beneficial microbes [2–8]. To balance the costs and benefits of these various microbes, individuals can increase microbial transmission by performing behaviours such as mouth-to-mouth regurgitations or consuming faeces [1], or decrease microbial transmission through behaviours such as avoiding sick individuals [9].

Understanding the effects of social transmission on microbiome similarity in free-living animals is challenging, however, due to confounding effects of kinship and shared environments [1]: offspring might acquire microbes from parents, and individuals that are highly associated will often share the same spaces, food and water. Such influences are hard to disentangle in observational field studies. A more powerful approach is to control these confounding factors experimentally, rather than statistically.

Here, we introduce individuals from distant sites and track their social interaction rates to assess the role of social interactions in shaping gut microbiome composition. We used the common vampire bat, *Desmodus rotundus*, a highly social species and obligate blood feeder. Due to this specialized diet, dietary variation plays a smaller role in explaining microbial diversity among vampire bats [10–12] compared to mammals with less specialized diets [13]. On the other hand, social transmission of microbes is likely a causal driver of microbial diversity in this species because female vampire bats hang in tight clusters and spend about 5% of their awake time grooming other individuals' wings or fur with their mouths [14]. In addition, females regurgitate blood to their offspring and to other highly associated adults that failed to feed [14–17]. This oral contact (mouth-to-body allogrooming and mouth-to-mouth regurgitated food sharing) provides channels for microbial sharing between individuals.

To assess evidence for group-level convergence in the vampire-bat social microbiome, we sampled the faecal and gut microbiome of captive-born vampire bats from six zoos, 15 female vampire bats from a wild colony in Belize and an ‘experimentally merged’ colony composed of 26 wild-caught vampire bats and five captive-born offspring sourced from three distant sites in Panama, then housed together in captivity for four months*.* In the experimentally merged colony, social networks of rates of clustering and oral contact predicted gut microbiome similarity, and faecal samples showed evidence for microbiome convergence over time.

## 2. Material and methods

### (a) Sample collection

To assess colony-level variation in faecal microbiomes, we obtained three to six faecal samples from vampire bats housed at six zoos: (i) North Carolina Zoological Park, Asheboro, North Carolina; (ii) Cincinnati Zoo and Botanical Garden, Cincinnati, Ohio; (iii) Dallas World Aquarium, Dallas, Texas; (iv) Memphis Zoological Garden and Aquarium, Memphis, Tennessee; (v) Aquarium and Rainforest at Moody Garden, Galveston, Texas; and (vi) Sedgwick County Zoo, Wichita, Kansas. In Belize, we collected faecal samples from 15 female bats (capture and fieldwork described previously [18]). In Panama, we individually sampled 26 wild-caught bats that were experimentally merged into one colony, as well as five captive-born offspring, for studies on social relationship formation (electronic supplementary material, table S2). We captured all bats from three distant wild roosts (120–340 km apart): six adult females, one juvenile female and two juvenile males from a cave at Lake Bayano, Panamá, 10 adult females from a hollow tree in Tolé, Panamá (including one bat that was not part of the merged colony) and eight adult females from a hollow tree in La Chorrera, Panamá. We then housed these bats together in an outdoor flight cage for four months as described previously [19]. We opportunistically took 85 faecal samples from isolated bats from 19 May 2019 to 16 October 2019. In all study colonies, faecal samples were smeared in duplicate on FTA cards (Whatman, GE Healthcare, sup. no. WB120055).

In the experimentally merged colony, bats were fed with cattle or pig blood from a meat processing plant that was chemically defibrinated with 44 g of sodium citrate and 16 g of citric acid per 19 l container. The blood was either refrigerated for up to 6 days or stored frozen, then thawed immediately before being provided to the bats. Nine bats developed a *Staphylococcus* infection during the study, which required administration of enrofloxacin and isolation from the rest of the colony from 21 July to 5 August. However, no faecal samples were collected after 13 July, and we also failed to detect any clear effect of this antibiotic on the diversity of final gut microbiome sampled on 15 October (mean and 95% CI of Simpson diversity: 19 untreated bats = 0.881 [0.874, 0.887], nine treated bats = 0.876 [0.868, 0.883]; *t*-test: *t* = 0.92, d.f. = 19.3, *p* = 0.4). For the final gut sample, bats from the experimentally merged colony were sacrificed using isoflurane to anaesthetize them (inhaled ≥ 5%) prior to rapid decapitation. Gut samples were collected from the distal colon and smeared on FTA cards.

### (b) Social network construction

In the merged colony, we recorded clustering and oral contact from video recorded by three infrared surveillance cameras (Foscam NVR Security System) for 6 h each day from 23 June 2019 to 4 August 2019, and from 11 August 2019 to 14 October 2019 (a total of 640 sampled hours), as described previously [19]. To measure clustering, all bats that were roosting in a contiguous group at the start of each half-hour were scored as associated. To measure rates of oral contact (which includes mouth-to-body or mouth-to-mouth contact), we measured the duration of any bout of licking that was at least 5 s in duration, noting the actor and receiver. Because mouth-to-mouth contacts were rare (never observed in 71% of pairs), we do not analyse them separately from mouth-to-body contacts.

We calculated undirected clustering networks using the simple ratio index in the R package asnipe [20], and undirected contact rates as the mean of the total duration of dyadic interaction bouts in both directions for each sample hour during which both bats were present in the flight cage. To reduce extreme skew in the edge weights for oral contact, we applied an inverse reciprocal transformation to (1+ oral contact rates). We scaled network matrices to standardize units to standard deviations across variables.

### (c) Microbiome sequencing

DNA was extracted from FTA cards in a designated pre-PCR BSL-2 laboratory using the Qiagen PowerSoil kit (Qiagen; cat. no. 47014). Every batch of 8–24 sample extractions included a negative control. Samples with discoloration after the wash buffer step in the protocol were eluted and re-bound to filter columns with binding buffer for one to three additional washes.

Genomic libraries were constructed using the Illumina DNA Prep kit (Illumina, cat. no. 20018705). Duplicate samples were randomly selected or combined for separate library builds for the Belize colony, the aggregated zoo populations and the experimentally merged colony (electronic supplementary material, table S1). Extraction negatives had negligible quantities of DNA as quantified by Qubit readings (ThermoFisher Scientific, cat. no. Q32851), and were combined to make per-population negative libraries. A separate library negative was also constructed. Overall, 145 samples, including samples, duplicates and listed negative controls, were sequenced on a NovaSeq 6000 S4 lane (Illumina), resulting in approximately 1.75 billion 150 bp paired-end reads. Because we used DNA extraction without reverse transcription, our microbiome analyses do not include RNA viruses.

### (d) Bioinformatics

Sequences from negative extraction and sequencing libraries were used to create population-specific filtering databases in addition to the human genome database provided by KneadData to filter all sample reads [21]. Paired-end reads were combined and taxonomically profiled using MetaPhlAn 3 [21]. MetaPhlAn 3 was run using default parameters, assigning taxonomy to genus/species based on unique nucleotide sequence markers and ignoring sequences that fail to match these markers [22]*.* We found high consistency in the duplicates from these initial profiles, varying less than 5% in read counts attributed to each taxa. Once initial analyses were complete, duplicates (both paired sets of reads) were combined with their partner for further analyses.

MetaPhlAn 3 identified 102 species-level taxa across all three populations [21]. Alignment data were analysed using the phyloseq R package [23–25]. To account for variable sequencing depth, we used a variance stabilized transformation, and assessed abundance in sample types and populations using DESeq2 [26]. Functional analysis of the filtered reads was done with HUMAnN3; hits were grouped by enzyme classes and split to taxonomic levels where possible [21]. We converted taxonomy-associated read data into Bray–Curtis distances, and defined ‘microbiome similarity’ as 1—Bray–Curtis distance.

### (e) Statistical inference

To test the simultaneous effects of clustering and oral contact networks on gut microbial similarity in the experimentally merged colony, we used multiple regression quadratic assignment procedure with double semi-partialing (MRQAP-DSP) from the asnipe R package [20,27]. To create all 95% confidence intervals shown in square brackets, we used percentile bootstrapping in the boot R package [28].

To test for evidence of convergence in similarity of faecal samples, we compared the similarity of initial opportunistic pre-merge faecal samples from five Lake Bayano bats on 19 May 2019 to later samples from other Lake Bayano bats and to later samples from three Tolé bats that were also sampled before and after the merge on 14 June 2019. We then calculated the change in similarity for the 25 pairs where both bats were sampled before and after the merge (10 pairs captured at the same wild roost and 15 pairs introduced in captivity). To test the prediction that faecal microbiomes converged for introduced pairs and diverged for same-roost pairs, we fit a general linear mixed effects model (MCMCglmm function and package using default priors) where fixed effects were time (days since initial sample, scaled), dyad type (same-roost versus introduced) and the interaction between time and dyad type. Both bats were entered as a multi-membership random effect. After an interaction was detected, we fit the same mixed model for both dyad types separately with time as the fixed effect. As an alternative approach, we also tested the interaction term using a permutation test; to get a permutation *p*-value, we fit the model with the same fixed effects (no random effects), then simulated the null hypothesis by randomizing the time differences within each dyad 1000 times to generate a distribution of expected values to compare with the observed.

## 3. Results

**Figure 1.**
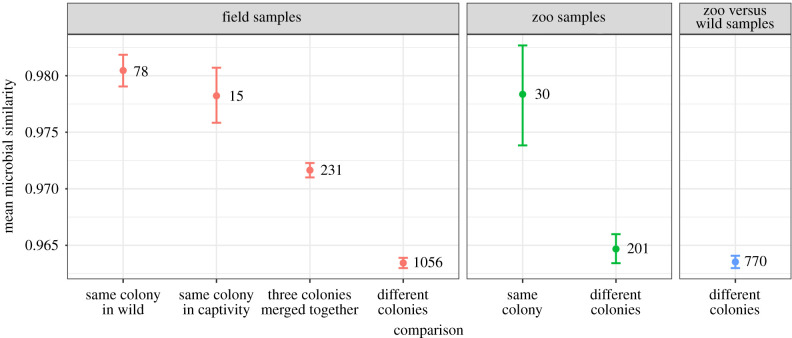
Mean faecal microbiome similarity between bats from the same or different colonies is consistent with social convergence. Microbiome similarity was measured for faecal samples of two bats from the wild (red), two bats from the zoo (green) or one zoo and one wild bat (blue). Mean microbiome similarity with bootstrapped 95% confidence intervals are shown for (left to right): 78 pairs of bats in the same wild colony (in Belize), 15 bat pairs in the same captive colony (two bats from Lake Bayano before introduction of other Panama colonies), 231 pairs of bats from three different colonies merged together (Lake Bayano, Tolé or La Chorrera), 1056 pairs of bats from two different wild colonies (Belize versus Panama), 30 pairs of samples from the same zoo colony, 201 pairs of samples from two different zoo colonies or 770 pairs of samples from one zoo bat and one wild bat.

Bats from the same colony had more similar faecal microbiomes than bats from different colonies (*β* = 0.55, *n* = 57 bats, *p* < 0.001; figure 1). The rank order of faecal microbiome similarity from high to low was: (i) bats from the same colony, (ii) bats from different colonies merged into one colony and (iii) bats from different colonies, with clear differences in similarity among all cases (figure 1).

Over four months together, all of the introduced Panama bats from distant sites engaged in clustering, and most engaged in oral contact (78%, electronic supplementary material, figure S1). The final gut microbiome similarity was predicted by rates of both oral contact and clustering, each when controlling for the other (MRQAP: oral contact *β* = 0.24, *n* = 27, *p* < 0.0001; clustering *β* = 0.28, *p* < 0.0001). Each effect also remained when controlling for whether the bats shared their capture site (clustering: *β* = 0.37, *p* < 0.001, *n* = 27, capture site: *β* = −0.30, *p* = 0.003; oral contact: *β* = 0.32, *p* < 0.001; capture site: *β* = −0.21, *p* = 0.07). After four months together, bats sourced from the same wild roost did not have more similar gut microbiomes than introduced bats (*β* = −0.17, *p* = 0.2), and we did not detect that the five mothers had more similar gut microbiomes to their own pups (mean similarity = 0.974 [0.966, 0.981]) compared to other pups (0.972 [0.968, 0.975]).

**Figure 2.**
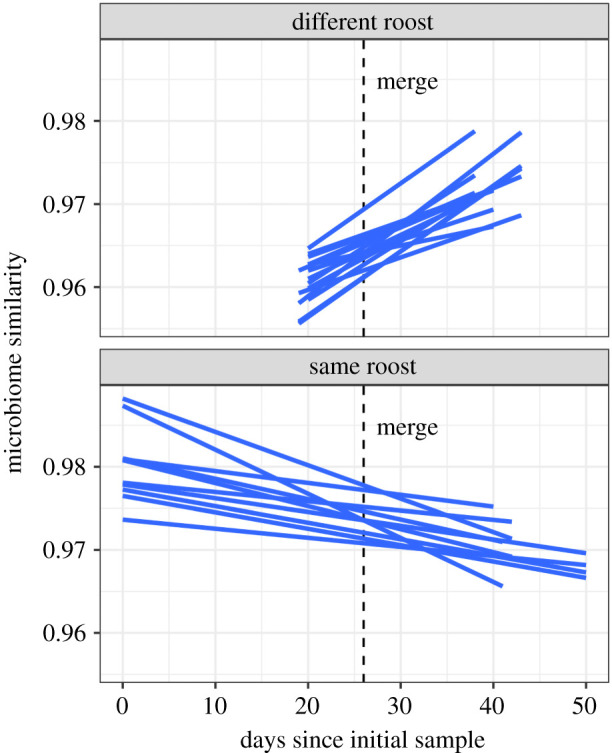
Evidence for convergence from opportunistic faecal samples from eight bats. Each regression line shows the change in microbiome similarity for one of 25 selected pairs of bats from different roosts (top) or the same roost (bottom). For each pair, the first bat was sampled soon after capture and prior to the colony merge (dashed line), while the second bat was sampled both before and after the colonies were merged. Bats from different roosts were captured 340 km apart. Regression lines are labelled by dyad in electronic supplementary material, figure S2.

All 25 pairs that were sampled both before and after the merge had faecal microbiome similarities that changed in the predicted direction (MCMCglmm: posterior estimate of interaction with 95% credible interval = −0.12 [−0.13, −0.09], pMCMC < 0.001, permutation test *p*-value < 0.001); they increased in the 15 introduced pairs (+0.08 [0.06, 0.10], pMCMC < 0.001), and decreased in the 10 same-roost pairs (−0.04 [−0.05, −0.03], pMCMC < 0.001, figure 2).

## 4. Discussion

Three lines of evidence from both our broader sampling and controlled experiment suggest social transmission of microbes in common vampire bats leads to a ‘social microbiome’ [1]. First, vampire bats from the same colony had more similar faecal microbiomes than bats from different colonies, in both captivity and in the wild (figure 1). Second, after bats from three wild colonies were introduced in captivity and housed together for four months, the identity of their original wild colony did not predict gut microbiome similarity; instead, gut microbiome similarity in the experimentally merged colony was predicted by rates of clustering and oral contact (electronic supplementary material, figure S1). Third, analysis of microbial similarity in opportunistic faecal samples from eight bats in the experimentally merged colony found that all 15 introduced pairs converged while all 10 same-roost pairs diverged (figure 2).

These findings corroborate previous studies of primates and rodents showing that social networks predict microbiome similarity after statistically controlling for environment and diet [6,29–41]. For example, laboratory mice housed together show convergence in their microbiomes [42,43]. A key advantage of our study is that we combined a controlled diet and environment with high-resolution interaction rates (sampling 6 h d^−1^ for four months) among both familiar individuals and individuals with no previous contact. Our findings confirm that direct horizontal social transmission of microbes is an important component of microbiome similarity [1]. While mammalian gut microbiomes can be seeded through vertical transmission at birth [44], these microbiomes are highly mutable [34–39,45]. Even in highly constrained communities, like the gut microbiomes of the common vampire bat, we still see identifiable variation across populations and over time.

A common challenge in microbiome studies, especially in novel host animals, is the inability to identify significant portions of the microbial community. In studies of social transmission of microbes, strain-level identification appears to present the most robust evidence for social behaviours transmitting microbes, yet this method is rare [46,47]. Increasing the depth and breadth of sequencing of a microbiome, as done here, is a useful step in providing sequence data for novel and less-characterized microbes, which can aid attempts to better track specific taxa that are socially transmitted. The high consistency of identified species in the microbiomes we observed within and across vampire bat colonies from different geographical locations and states of captivity might be a result of their obligate and specialized diet, but many of our reads (approx. 76%) could not be assigned to any known taxa, showing yet unknown complexities in the common vampire bat's microbiome.

## Data accessibility

The datasets and R code supporting this article have been uploaded to Figshare [48]. Sequence data are available through the NCBI SRA under BioProject PRJNA739024.
